# Use of the Comet-FISH Assay to Compare DNA Damage and Repair in p53 and hTERT Genes following Ionizing Radiation

**DOI:** 10.1371/journal.pone.0049364

**Published:** 2012-11-07

**Authors:** Declan J. McKenna, Bernadette A. Doherty, C. Stephen Downes, Stephanie R. McKeown, Valerie J. McKelvey-Martin

**Affiliations:** Biomedical Sciences Research Institute, University of Ulster, Coleraine, Northern Ireland, United Kingdom; The Chinese University of Hong Kong, Hong Kong

## Abstract

The alkaline single cell gel electrophoresis (comet) assay can be combined with fluorescent *in situ* hybridisation (FISH) methodology in order to investigate the localisation of specific gene domains within an individual cell. The number and position of the fluorescent signal(s) provides information about the relative damage and subsequent repair that is occurring in the targeted gene domain(s). In this study, we have optimised the comet-FISH assay to detect and compare DNA damage and repair in the p53 and hTERT gene regions of bladder cancer cell-lines RT4 and RT112, normal fibroblasts and Cockayne Syndrome (CS) fibroblasts following γ-radiation. Cells were exposed to 5Gy γ-radiation and repair followed for up to 60 minutes. At each repair time-point, the number and location of p53 and hTERT hybridisation spots was recorded in addition to standard comet measurements. In bladder cancer cell-lines and normal fibroblasts, the p53 gene region was found to be rapidly repaired relative to the hTERT gene region and the overall genome, a phenomenon that appeared to be independent of hTERT transcriptional activity. However, in the CS fibroblasts, which are defective in transcription coupled repair (TCR), this rapid repair of the p53 gene region was not observed when compared to both the hTERT gene region and the overall genome, proving the assay can detect variations in DNA repair in the same gene. In conclusion, we propose that the comet-FISH assay is a sensitive and rapid method for detecting differences in DNA damage and repair between different gene regions in individual cells in response to radiation. We suggest this increases its potential for measuring radiosensitivity in cells and may therefore have value in a clinical setting.

## Introduction

The comet-FISH assay is a method that allows DNA damage and repair to be detected in specific gene regions relative to the overall genome [1]. It has been used in several studies that have successfully localised DNA damage within comets using both chromosome and gene-specific probes (reviewed in [2]). The ability to obtain this type of information would be useful because this assay could benefit many areas of clinical investigation by providing valuable information about the intrinsic DNA characteristics of individual cells and their responses to various external factors, such as radiation, chemicals and drugs. This information would prove particularly relevant in the diagnosis, prognosis and treatment of cancer by allowing analysis of tumour cells, since the repair of important gene regions is integral in determining individual patient response to therapy [3]. Indeed, the Comet assay in its various forms is an attractive candidate for a predictive test for radiosensitivity in a clinical setting [4]. The ability to predict the radiosensitivity of individual tumours would represent a major step forward in radiation biology, since there is still no definitive way of predicting whether an individual patient will respond to radiotherapy or not.

A number of different techniques have been developed to address this problem with varying degrees of success, such as the SF2 clonogenic survival assay [5], potential doubling time (Tpot) of the tumour [6], tumour hypoxia measured by pO2 [7], the percentage of apoptotic or viable cells [8], index of thymidine and BudR labelling [9], immunohistochemical detection of specific proteins [10] and microarray technology [11]. However, the comet assay offers many advantages over these, since it is a relatively simple and inexpensive technique, which requires only a few cells and results can be obtained within a matter of hours. Encouragingly, several studies have shown that the comet assay is a reliable and comparable alternative to the time-consuming clonogenic survival assay, currently considered the gold standard method for predicting tumour sensitivity [12]–[15]. However, for the comet assay to gain widespread acceptance for this application, more studies are required to demonstrate what sort of unique information it can provide. If information about damage and repair in specific gene regions could also be obtained by employing the comet-FISH version of the assay, this would increase the diagnostic and prognostic potential of this technique as a routine application in the clinical laboratory.

With this in mind, we have used the comet-FISH assay to simultaneously probe two gene regions in order to compare DNA damage and repair in different gene regions within the same cell. We have targeted the p53 (17p13.1) and hTERT (5q15.33) gene loci since these genes are known to have different transcriptional activities and should therefore exhibit differences in DNA repair efficiency in our assay. The p53 gene is actively transcribed [16], is induced by γ-radiation [17] and is known to be preferentially repaired in comparison to other genes [18], whereas the hTERT gene is transcriptionally inactive in normal cells but activated in the majority of tumour cells [19], [20]. hTERT codes for human telomerase reverse transcriptase, the catalytic subunit of the enzyme telomerase and transcriptional up-regulation of hTERT has been shown to occur in >85% of human neoplasms clearly identifying it as a potentially useful biomarker for tumourigenesis [20]. This makes both genes ideal candidates for testing in the comet-FISH protocol.

Previous studies in our laboratory have optimised the comet-FISH assay and shown that the p53 gene region in two bladder cancer cell lines was more rapidly repaired than the overall genome following treatment with both γ-irradiation [21] and the DNA cross-linking agent mitomycin C [22]. We hypothesised that this preferential repair might be a reflection of transcription-coupled repair (TCR) occurring within the cell and proposed that the comet-FISH assay offered considerable potential for further study of gene-region specific repair. Therefore, in this current report, we build upon these previous studies and compare γ-radiation-induced DNA damage and repair between the p53 and hTERT gene regions in bladder cancer cell lines (RT4 & RT112), normal fibroblast cells (GM38) and in two Cockayne Syndrome fibroblast cell lines (CSA & CSB), which are defective in TCR. Since we are employing alkaline conditions in the Comet-FISH assay, we will be measuring a combination of frank strand breaks and other alkaline labile sites that give rise to secondary strand breaks. Therefore, when we make reference to DNA repair, it is of these particular DNA lesions we are considering. We demonstrate that this assay can successfully detect differences in DNA repair between two separate gene regions following radiation, thereby establishing it as a technique which offers great potential for monitoring the gene-specific response to DNA damage in individual cells and populations.

**Figure 1:**
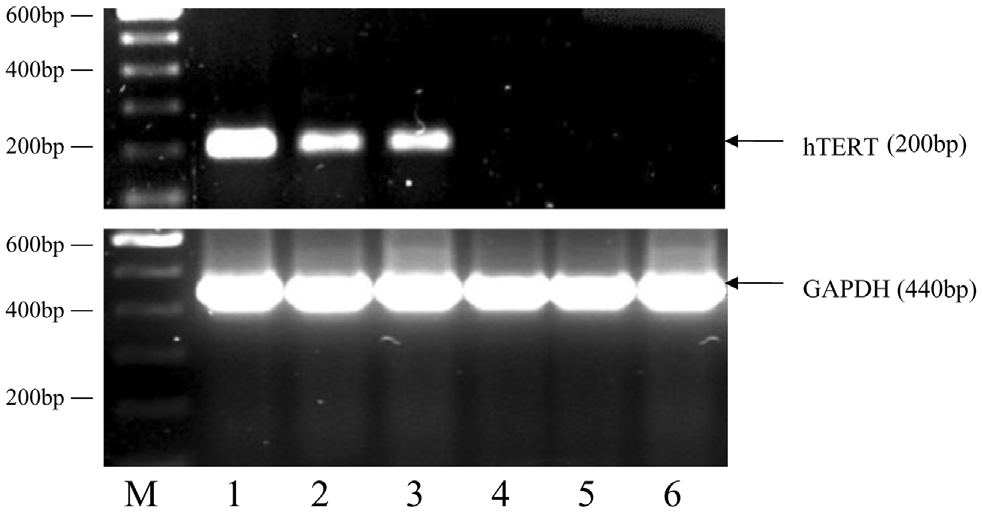
hTERT Expression in Cell Lines. Top image shows hTERT expression detected at 200 bp in a Werner syndrome cell line (positive control) immortalised for hTERT (Lane1). hTERT expression is also detected in the bladder cancer cell lines RT112 and RT4 (Lanes 2 and 3 respectively). No hTERT expression was found in the normal GM38 fibroblast cell line (Lane 4) or in the two Cockayne Syndrome cell lines, CSA and CSB (Lanes 5 and 6 respectively). Bottom image shows the expression of housekeeping control gene GAPDH at 440 bp in all six cell lines (Lanes 1–6). (Lane M, 100 bp size marker.).

## Materials and Methods

### Cell Lines and Cell Culture

The normal, CSA and CSB fibroblast cell lines used in this study are commercially available from the Human Genetic Mutant Repository (Coriell Institute, Camden, NJ). The normal fibroblast cells (GM38) [23] were cultured in Eagle’s minimum essential medium (EMEM), supplemented with 20% foetal bovine serum (FBS), 4% essential amino acids, 2% non-essential amino acids and 1% penicillin-streptomycin. The CS fibroblast cell lines used in this study, CSA (GM01856) and CSB (GM00739), were maintained in EMEM, supplemented with 15% FBS and containing 1% penicillin-streptomycin. RT112 bladder cancer cell line [24] was obtained from the European Collection of Cell Cultures (ECACC) (Salisbury, UK) and RT4 [25] bladder cancer cell line was obtained from the American Tissue Culture Collection (ATCC) (Rockville, MD). RT112 cells were cultured in Minimum Essential Medium (MEM), supplemented with 10% FBS and containing 1% penicillin-streptomycin. RT4 cells were cultured in McCoy’s 5A medium, supplemented with 10% FBS and 1% penicillin-streptomycin. Werner syndrome (WS) [26] cells obtained from the Coriell Cell Repository (Camden, NJ) were maintained in MEM, supplemented with 15% FBS and 1% penicillin-streptomycin. This cell line has been immortalised with hTERT and was used in our PCR experiments as a positive control for this gene (Simpson, unpublished). The GM38 cell line [27], RT4 cell line [28] and both CS cell lines (Alan Lehman, personal communication) all actively express wild type p53, whereas the RT112 cell line contains a point mutation at codon 248 resulting in an Arg-Gly amino acid change and therefore expresses mutant p53 [29].

**Figure 2:**
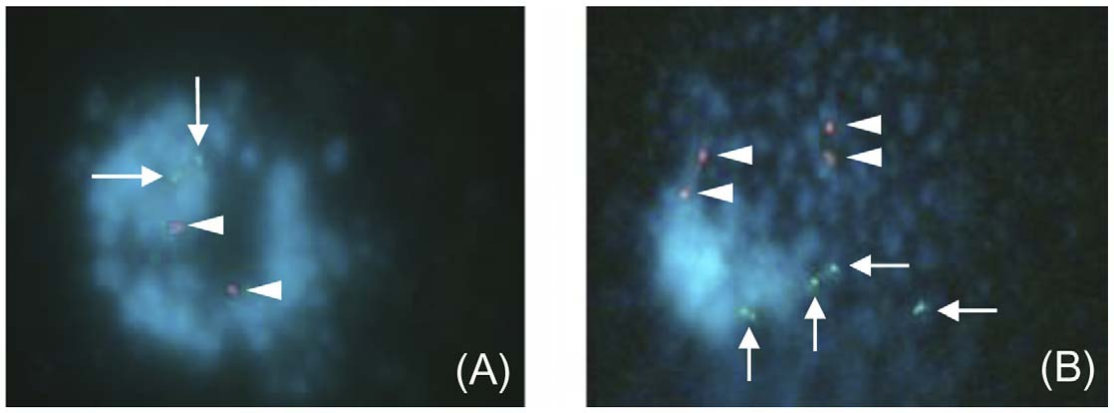
Representative images of comet-FISH cells. A. An untreated RT112 cell displays hardly any DNA damage evidenced by a relatively intact comet head and the absence of a comet tail. Two pink p53 (arrowheads) and two green hTERT (arrows) hybridisation spots are visible in the intact comet head. **B.** Immediately following 5Gy γ-irradiation, a large, dispersed comet tail is visible, indicating a significant amount of overall DNA damage. Cells display several p53 and hTERT hybridisation spots in both the comet head and tail, indicating that some radiation-induced strand breaks have occurred within, or close to, both gene regions.

### RT-PCR Analysis of hTERT Gene Expression

Total RNA was extracted from the CSA, CSB, GM38 and WS cell lines using the RNeasy midi kit (Qiagen, Crawley, UK) according to the manufacturer’s instructions. The quantification and quality of RNA was determined spectrophotometrically and by 1% agarose gel electrophoresis. Complementary DNA (cDNA) was obtained for each cell line using a Superscript^TM^II RNase H- Reverse Transcriptase Kit (Invitrogen, UK) and PCR performed. The specific primers used for PCR amplification of hTERT were 5′-CTCACCTTCAACCGCGG-3′(sense) and 5′-TTGCTGATGAAATGGGAGCT-3′ (antisense), generating a 200 bp amplicon (genbank accession number  =  NM198253). For GAPDH, used as a positive control, the primers were 5′-ACCCCTTCATTGACCTCAACTACA-3′ (sense) and 5′ TACTGGTGTCAGGTACGGTAGTGA-3′ (antisense), generating a 440 bp amplicon. For hTERT expression, a sample from a WS patient immortalised with hTERT was used as a positive control. Reaction conditions were 31 cycles of denaturation at 94°C for 45 seconds, annealing at 60°C for 45 seconds and extension at 72°C for 90 seconds for hTERT and 30 cycles of denaturation at 95°C for 1 minute, annealing at 56°C for 1 minute and extension at 72°C for 5 minutes for GAPDH. The amplified PCR products were separated by electrophoresis on a 1% agarose gel and visualised by staining with ethidium bromide (Sigma). The expression of hTERT mRNA relative to GAPDH mRNA was determined using gel analysis software Scion Image (Scion, Frederick, MD, USA) to measure the density of respective DNA bands.

**Table 1:** Table shows data generated by comet-FISH assay for all 5 cell lines at 0, 15, 30 & 60 minutes following 5Gy radiation.

		Control	0	15	30	60
**RT4**	% Tail DNA	6.75±0.88	47.16±1.87	34.82±2.04	30.33±4.6	24.7±4.94
	% Cells showing DNA Damage	38±4	100±0	99±1	87±7.06	80±8
	% Cells with p53 Tail Spots	29±1	100±0	69±5	67±3	55±3
	% Cells with hTERT Tail Spots	71±5	100±0	91±3	89±3	83±1
**RT112**	% Tail DNA	9.9±0.65	45.89±0.35	31.25±2.23	23.19±3.64	24.4±0.99
	% Cells showing DNA Damage	47±1	100±0	92±2	81±5	72±0
	% Cells with p53 Tail Spots	22±2	75±5	54±2	37±3	29±1
	% Cells with hTERT Tail Spots	65±1	91±7	86±0	76±8	71±1
**GM38**	% Tail DNA	6.10±0.45	30.78±1.17	28.71±0.09	21.22±1.24	17.05±0.62
	% Cells showing DNA Damage	47±5	99±1	98±0	91±5	78±4
	% Cells with p53 Tail Spots	3±2.16	90±2	54±6	46±5.5	50±2
	% Cells with hTERT Tail Spots	16±2	82±2	83±3	77±3	73±3
**CSA**	% Tail DNA	6.41±0.3	44.14±2.47	33.44±4.33	21.94±2.28	23.43±4.48
	% Cells showing DNA Damage	38±4	98±2	97±1	90±2	90±6
	% Cells with p53 Tail Spots	25±1	90±4	84±4	85±7	77±3
	% Cells with hTERT Tail Spots	41±1	92±2	91±1	88±4	86±6
**CSB**	% Tail DNA	6.28±0.55	44.90±0.36	28.99±0.9	26.63±2.11	19.08±0.59
	% Cells showing DNA Damage	39±1	99±1	91±5	81±3	89±3
	% Cells with p53 Tail Spots	12±4	97±3	87±1	88±4	82±0.02
	% Cells with hTERT Tail Spots	26±4	98±0.02	84±4	90±2	76±4

Each measurement is the mean ± SEM of two independent experiments, representing 100 cells in total.

**Figure 3:**
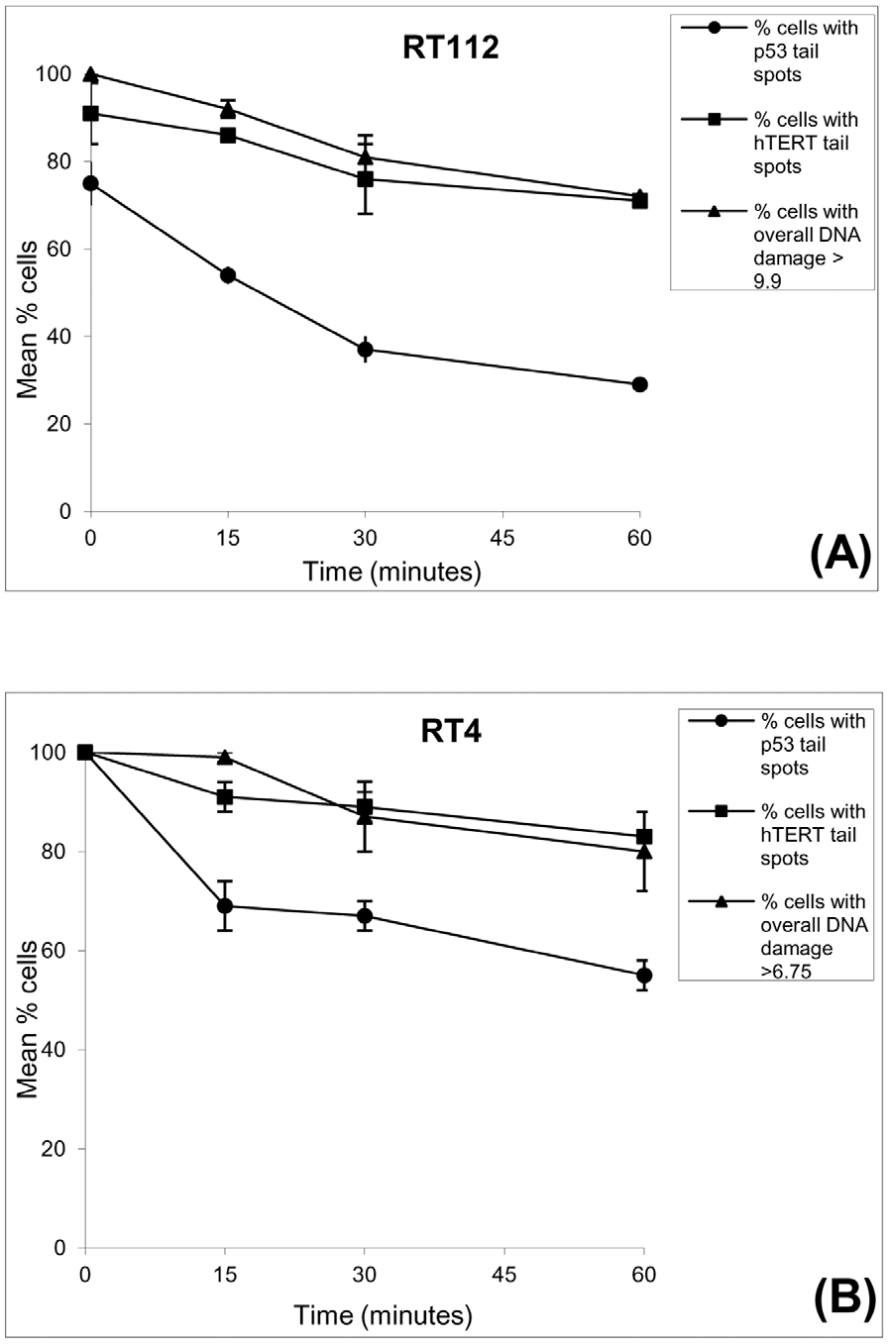
Comparison of repair of overall genomic DNA damage and of the p53 and hTERT gene regions in (A) RT112 cells (B) RT4 cells following 5Gy radiation. Each graph shows the mean % of cells showing γ-radiation-induced damage (▴). A cell was considered to have γ-radiation-induced DNA damage if its mean % DNA measurement had a greater value than the mean % DNA value for control cells for that cell line (control values shown in key on each graph). The mean % of cells showing p53 (•) and hTERT (▪) hybridisation spots in the comet tail is also shown for each cell line. For both cell-lines the rate of repair of overall genomic DNA and hTERT is similar (as shown by slope of lines), whereas the rate of repair of p53 is significantly faster during the first 15 minutes (P<0.05 for both cell lines). Each data point represents the mean ± SEM of two independent experiments.

### Comet-FISH Assay

#### Gel preparation and comet assay

The alkaline comet assay was performed according to a standard protocol of McKelvey-Martin *et al* [2]. Cells were harvested and washed twice in 10 ml phosphate buffered saline (PBS). Cell viability was assessed using the trypan blue exclusion method. In all experiments, cell viability was >99%. One millilitre aliquots of the cell suspensions in Ca^2+^ and Mg ^2+^ free PBS, at a concentration of 2×10^5^ cells/ml were pipetted into eppendorf tubes and centrifuged at 1500 rpm for 5 minutes at 4°C. Meanwhile, Dakin fully frosted microscope slides (Labcraft, UK) were each covered with 100 µl of 0.6% normal-melting-point agarose (prepared in Ca^2+^ and Mg^2+^ free PBS) at 37°C. A 22×22 mm cover slip was placed on top and the slide was kept on ice until the agarose had solidified. Low-melting-point agarose (1.2%) was mixed in a 1∶1 ratio with repair medium (growth medium containing 20% FBS), and 80 µl of this mixture was used to resuspend each pellet of cells. After the cover slip was gently removed, the cell/agarose suspension was quickly pipetted onto the first agarose layer, the cover slip was replaced, and the slide was left on ice to solidify the agarose. Following removal of the cover slips, 5Gy γ-irradiation (at a rate of 2cGy/s, using a Cs^137^ source) was administered and the slides were either (i) immediately placed in lysis solution (2.5 M NaCl, 100 mM Na_2_EDTA, 10 mM Tris, pH 10, with 1% Triton X-100 added) for 1hour at 4°C or (ii) the slides were immersed in the appropriate growth medium at 37°C for 15, 30 and 60 minutes before placing in lysis solution for 1 hour at 4°C. All slides were then placed in a horizontal gel electrophoresis tank filled with fresh chilled electrophoresis buffer (300 mM NaOH, 1 mM Na_2_EDTA, pH >13) to a level of approximately 0.25 cm above the slides. They were left for 20 minutes to allow DNA unwinding to occur before electrophoresis at 25 V (0.66 V/cm) and 300 mA for 20 minutes. Slides were then neutralised by 3 X 5 minute washes in 0.4 M Tris, pH 7.5, followed by a 5 minute wash in 2X SSC (3 M saline sodium citrate; 0.3 M sodium citrate, pH 5.3). The slides were then drained and subsequently dehydrated in an ascending series of ethanol solutions (70%, 85%, 100% for 2 minutes each) and air-dried.

**Figure 4:**
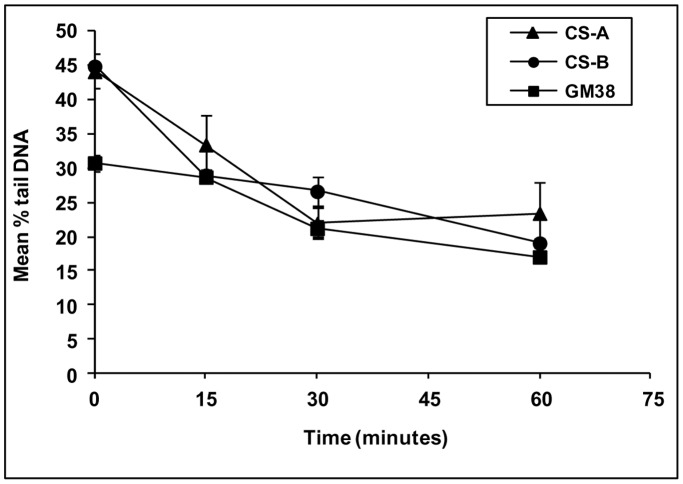
Repair of overall DNA damage in fibroblast cell lines measured by comet-FISH. Graph shows DNA damage and repair following the exposure to γ-radiation of normal GM38 fibroblasts and Cockayne Syndrome cell lines CSA and CSB. DNA damage is measured using mean % Tail DNA. Error bars shown are ±95% confidence limits.

#### Preparation of probes and hybridization

FISH was performed on prepared comet slides using a mix of 2 probes: (i) A locus-specific identifier (LSI) Spectrum-Orange-labelled p53 DNA probe, comprised of randomly sheared 50–140 bp lengths of DNA covering approximately a 145 kb region, including the 20 kb p53 locus (17p13.1) (Vysis, Surrey, UK); (ii) A LSI Spectrum-Green-labelled hTERT DNA probe, spanning a 180 kb region including the 40 kb hTERT locus (5q15.33) (Q-Biogene, UK). For each individual slide, a hybridisation mixture containing equal concentrations of the p53 probe and the hTERT probe was added and a 22 mm × 22 mm coverslip was placed on top. Co-denaturation of both target DNA and probe DNA was performed at 80°C for 2 minutes. Hybridisation of both probes took place simultaneously at 37°C for 16 hours in a dark, humidified chamber.

**Figure 5:**
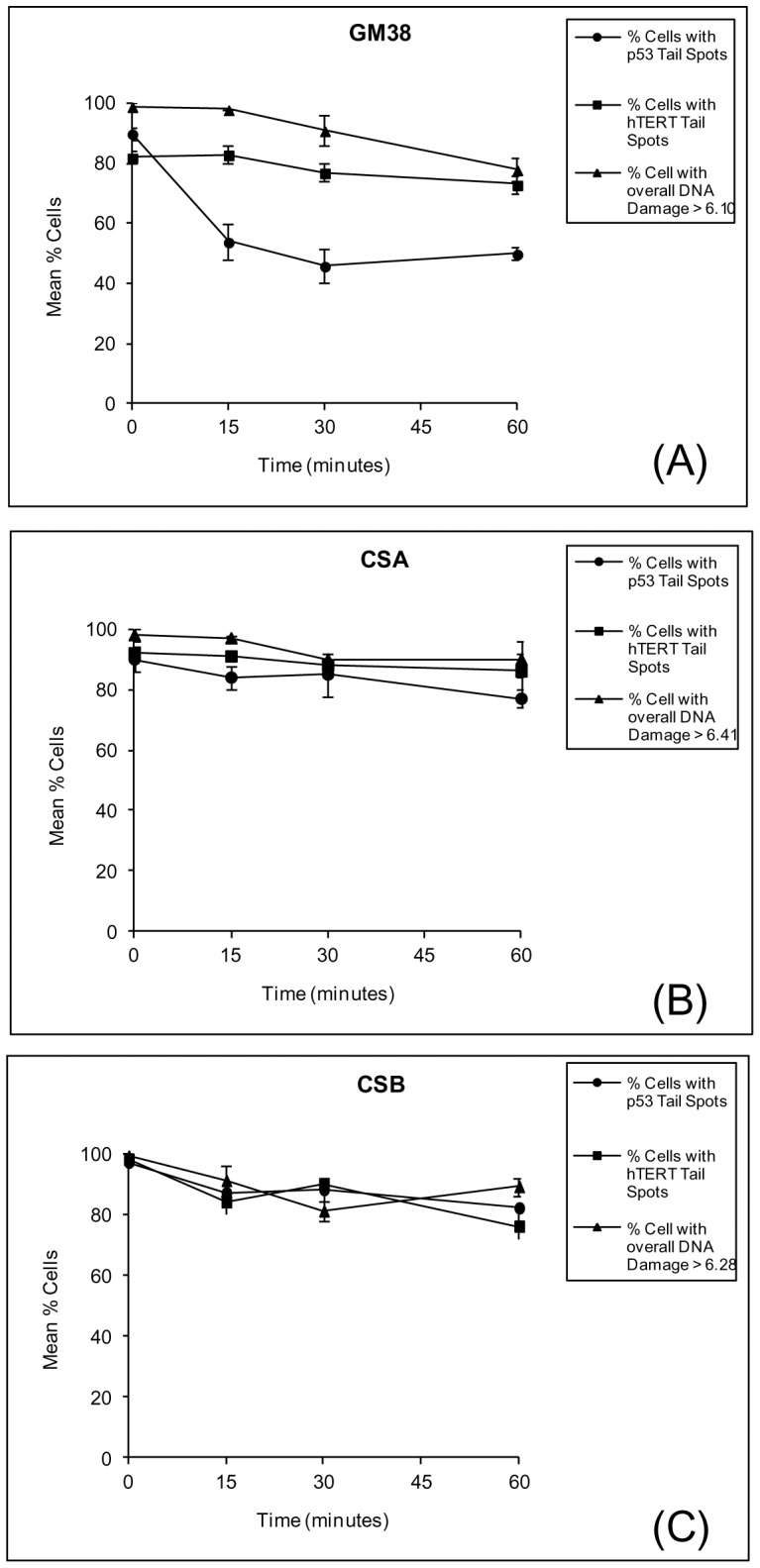
Comparison of repair of overall genomic DNA damage and of the p53 and hTERT gene regions in (A) GM38 cells (B) CSA cells (C) CSB cells following exposure to 5Gy γ-radiation. Each graph shows the mean % of cells showing γ-radiation-induced damage (▴). A cell was considered to have γ-radiation-induced DNA damage if its mean % DNA measurement had a greater value than the mean % DNA value for control cells for that cell line (control values shown in key on each graph). The mean % of cells showing p53 (•) and hTERT (▪) hybridisation spots in the comet tail is also shown for each cell line. In GM38 fibroblasts, the rate of repair of overall genomic DNA and hTERT is similar (as shown by slope of lines), whereas the rate of repair of p53 is significantly faster during the first 15 minutes (p<0.001). However, in CSA and CSB fibroblast, the rapid repair of p53 is not apparent and all three repair rates are similar with no significant differences observed between measurements. Each data point represents the mean ± SEM of two independent experiments.

**Figure 6:**
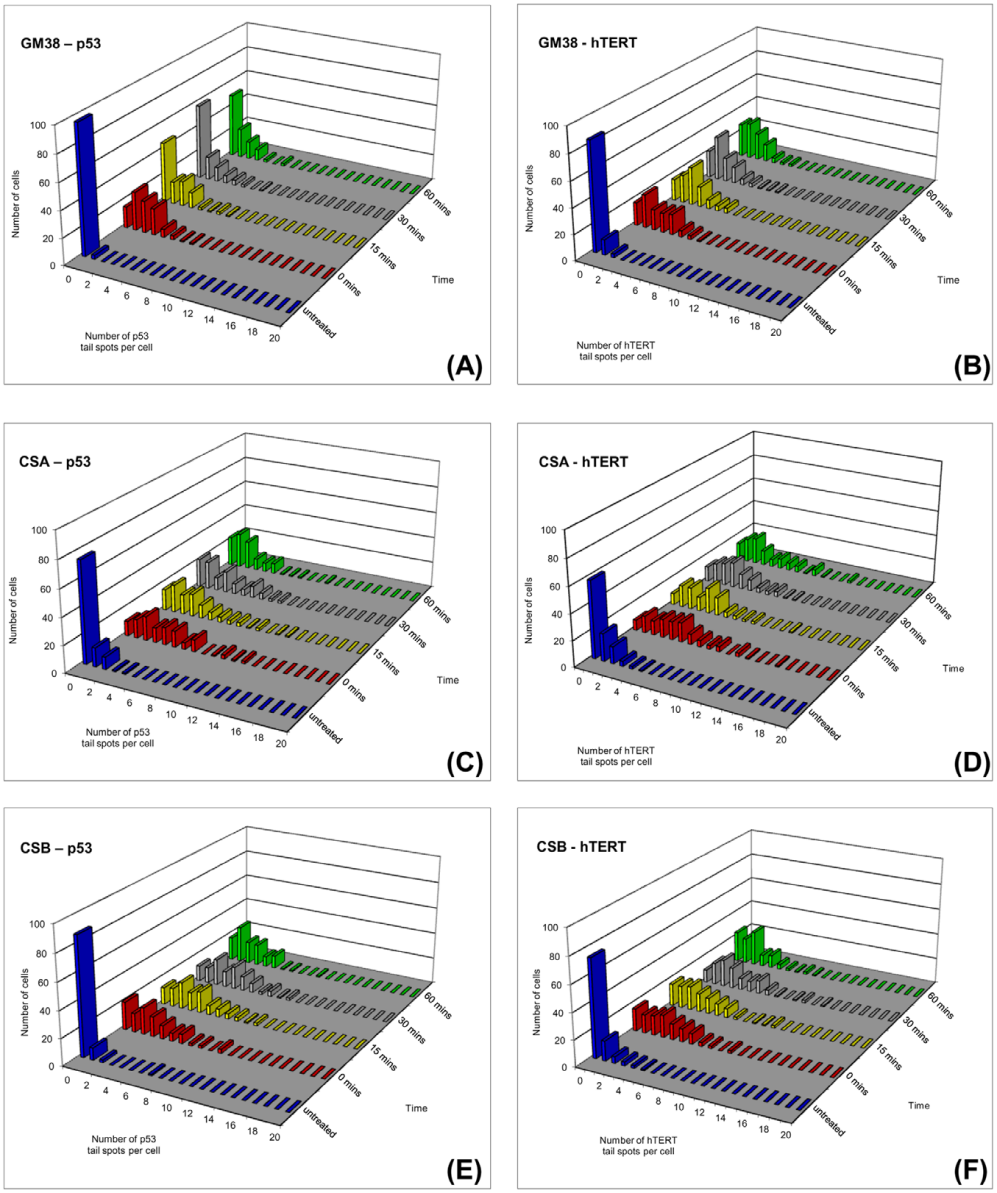
Frequency distribution analysis of p53 and hTERT hybridisation spot number at each repair time point following 5Gy γ-irradiation of fibroblasts. Each plot shows the number of p53 or hTERT tail spots per fibroblast in untreated cells and 0, 15, 30 and 60 minutes following irradiation. GM38 cells: (A) p53 (B) hTERT; CSA cells: (C) p53 (D) hTERT; CSB cells: (E) p53 (F) hTERT. For each cell line, the number of hybridisation signals detected in the tail is increased immediately after irradiation (t = 0). As repair occurs in GM38 fibroblasts, the number of cells displaying no p53 hybridisation spots in the comet tail is increased (see first bar in distribution profile). By contrast, the distribution profile of hTERT hybridisation spots remains relatively unaffected over the repair time. In CSA and CSB fibroblasts, no significant difference in the distribution profile is observed for either p53 or hTERT over the repair time-points.

### Post-hybridisation and Counterstaining

Following hybridisation, the slides were placed in a solution of 50% formamide and 2X SSC for 10 minutes at 45°C. Once in the solution, the slides were gently agitated to detach the coverslips. This wash was repeated three times, followed by a 10 minute wash in 2X SSC at 45°C and a 5 minute wash in 2X SSC containing 0.1% Igepal (Sigma). Slides were left to air dry for 30 min before being counterstained with 16 µl DAPI in antifade mounting solution (Vysis, UK). Subsequently, the slides were left in the dark at 4°C for no longer than 2 hours prior to observation. All experiments were carried out under yellow light to prevent additional DNA damage by natural light. All reagents were purchased from Sigma, Poole, UK unless otherwise indicated.

### Comet-FISH Analysis

Observations were made at a final magnification of x600 (Nikon x60 Fluor lens) using an epifluorescence microscope (Olympus BH2); equipped with Hitachi KP571 CCD camera interfaced through a Matrox IP8 board using Hewlett Packard Super VGA and Kromascan software (Andor Technology, UK). A triple bandpass filter set (Chroma HiQ) tuned for: DAPI (excitation 370 nm, emission 450 nm, bandwidth 20 nm), spectrum orange (excitation 560 nm, emission 590 nm, bandwidth 60 nm) and spectrum green (excitation 547 nm, emission 572 nm, bandwidth 30 nm), was utilised for comet-FISH. This enabled the simultaneous detection of: DAPI (overall genome damage), spectrum orange (p53 gene region) and spectrum green (hTERT gene region) labels. In addition to the number and position of p53 and hTERT hybridisation spots within each comet being noted, standard comet parameter measurements including tail moment and % tail DNA were also recorded for each comet. Comet analysis was performed using the Komet 5.0 digital imaging system (Andor Technology, UK), which measures a wide range of densitometric and geometric parameters for each comet. The primary measurement used in this study was % Tail DNA. One slide (50 cells) was analysed from each dose point. Two independent experiments were conducted to generate each data point (100 cells). Mean values for each measurement were generated standard error of the mean (SEM) was calculated from the standard deviation. Student t-test was used to generate statistics.

## Results

It has previously been established that the p53 gene is actively expressed in the RT4, RT112, CSA, CSB and GM38 cell lines used in our studies [27]–[29]. However, since we were not aware of the transcriptional activity of the hTERT gene in our cell lines, we measured the hTERT gene expression using RT-PCR. Figure 1 demonstrates that the hTERT PCR product was clearly detected in WS cells (positive control) and the RT112 and RT4 tumour cell lines, but not in the three fibroblast cell lines. These results agree with the findings of de Kok *et al* [19] and Abdul-Ghani *et al* [30] who demonstrated high levels of hTERT expression in RT112 and RT4 tumour cell lines but not in normal cells.

Representative images of cells processed in the comet-FISH assay demonstrate the typical appearance of cells when viewed through the microscope (Figure 2). By recording standard comet measurements, such as % Tail DNA, as well as the number and location of p53 and hTERT hybridisation signals for each cell, we could generate data at each time-point measured for all 5 cell lines (Table 1). The fluorescent signals were referred to as ‘head spots’ or ‘tail spots’, depending on their location within the comet.

We first focused our attention the γ-irradiation-induced DNA damage and repair in RT112 and RT4 bladder cancer cells. To measure overall DNA repair in the cells following 5Gy γ-irradiation, the mean % Tail DNA value was recorded for each time point (Table 1). A cell was considered to have radiation-induced DNA damage if its mean % tail DNA measurement had a value greater than the mean % tail DNA value for untreated cells. Immediately following irradiation, 100% of RT112 cells exhibited radiation-induced DNA damage as expected (Figure 3a). As the repair time increased, the number of RT112 cells showing damage decreased slowly, but steadily, to 72% by 60 minutes. hTERT tail spots were observed in 91% of RT112 cells immediately after irradiation, and this was repaired with a very similar rate to the overall genome with 71% of cells showing hTERT tail spots at 60 minutes. p53 tail spots were observed in 75% of RT112 cells immediately following irradiation. This was significantly reduced to 54% over the first 15 minutes of repair (p<0.05) and was further reduced by 30 minutes to 37%. Thereafter, a similar repair rate to hTERT and the overall genome was observed from 30–60 minutes.

Similar results were demonstrated by the RT4 cells (Table 1 and Figure 3b). Immediately following irradiation, 100% of RT4 cells showed radiation-induced DNA damage, and p53 and hTERT tail spots. As repair time increased, the % of cells showing overall DNA damage decreased at a slow, but steady rate to 80% at 60 minutes; a similarly slow rate of reduction to 83% by 60 minutes was found for cells with hTERT tail spots. Once again, the % of cells with p53 tail spots decreased significantly within the first 15 minutes to 69% (p<0.05). From 15–60 minutes, the rate of reduction was similar to that for hTERT and the overall genome.

Having identified preferential repair of the p53 gene region in tumour cells we then investigated if this phenomenon could be replicated in normal and repair deficient fibroblasts. To establish that the comet assay could measure overall DNA repair in these cell lines following γ-irradiation, we confirmed that a decrease in the mean % tail DNA measurements over the 60 minutes for all cell lines was apparent, thereby indicating active overall DNA repair (Table 1 and Figure 4). Greater initial damage, as measured by % Tail DNA, was observed in both CS cell lines compared to the GM38 cell line, since they contain intrinsically higher levels of baseline DNA damage. Similar results were obtained using Tail Moment results (data not shown).

We then wanted to see if the defective TCR in CS lines could be detected by comet-FISH. Figure 5 shows the repair of the overall genome in each fibroblast cell line as well as repair in the p53 and hTERT gene regions. For normal GM38 fibroblasts (Figure 5a), immediately following irradiation, 99% of cells exhibited significant radiation-induced damage. The majority of cells also showed damage in the p53 gene region (90% cells show p53 tail spots) and the hTERT gene region (82% cells show hTERT tail spots). The mean % of cells showing radiation-induced damage slowly decreased to 78% by 60 minutes. The repair of the hTERT gene region followed a similarly slow decrease with 73% of cells showing hTERT tail spots after 60 minutes. However, the mean % of cells with p53 tail spots was significantly reduced within 15 minutes to 54% (p<0.001). From 15 to 60 minutes, this measurement showed little significant reduction and followed a similar repair rate to hTERT and the overall genome.

A different trend was noted in both CS cell lines. Immediately following irradiation, 98% of CSA cells exhibited radiation-induced DNA damage and this decreased only slightly to 90% over 60 minutes (Figure 5b). The most notable difference from normal fibroblasts was the lack of change in the number and location of p53 hybridisation spots over the 60 minutes. Immediately following irradiation, p53 tail spots were observed in 90% of CSA cells, reducing to 77% after 60 minutes. Likewise, 92% of CSA cells displayed hTERT tail spots immediately after irradiation, reducing to 86% at 60 minutes. The CSB cell line demonstrated similar results to CSA (Figure 5c). Immediately following irradiation, 99% of CSB cells showed induced DNA damage, and all of these also exhibited p53 and hTERT tail spots. As repair time increased, the % of cells showing overall DNA damage decreased slightly to 89% at 60 minutes. As with CSA cells, the % of cells with p53 tail spots decreased slowly with 82% of CSB cells showing p53 tail spots after 60 minutes repair. Similarly, the percentage of cells showing hTERT tail spots was only slightly reduced from 98% to 76% over 60 minutes.

Recording the number and location of hybridisation spots also gives information about the extent of DNA damage and repair, since breaks within the probed target DNA can result in an increase in spot number, whilst a reduction in spot number may indicate a rejoining of strand breaks. Figure 6 shows the frequency distribution analysis of p53 and hTERT hybridisation spot number per tail in each fibroblast cell line at each repair time-point following treatment with 5Gy γ-irradiation. As expected, the majority of untreated GM38 cells have zero p53 or hTERT tail spots (Figure 6a and 6b). Following γ-irradiation (t = 0), the majority of cells display hybridisation signals for both probes in the comet tail, with some showing as many as six or eight spots. This indicates that breaks within, or near, the probed regions have occurred, and this broken DNA has migrated into the comet tail. Within 15 minutes, the number of cells displaying no p53 hybridisation spots in the comet tail is increased (see first bar in distribution profile). This indicates a rejoining of strand breaks in this gene region has occurred in a significant number of cells. By contrast, the distribution profile of hTERT hybridisation spots remains relatively unaffected over the repair time, indicating that repair of strand breaks is not as efficient in this region.

In CSA (Figure 6c and 6d) and CSB (Figure 6e and 6f) fibroblasts, no significant difference in the distribution profile is observed for either p53 or hTERT hybridisation signals. As with GM38 fibroblasts, irradiation increases the number of hybridisation signals for each probe, with the majority of cells in each population displaying several hybridisation spots in the comet tail (t = 0). Over the repair time, however, no clear differences are exhibited in either cell line between the distribution profiles of hybridisation signals. This demonstrates that the repair of stand breaks in the p53 gene region evidenced in GM38 fibroblasts is lost in these cells.

## Discussion

The comet-FISH assay enables us to assess both overall genomic repair (by measurement of the standard comet parameters) and gene region specific repair (by analysis of hybridisation spot location and number) in individual cells. In the current study, our results demonstrate that the p53 gene region is more rapidly repaired than the hTERT gene region in the bladder cancer cell lines RT4 and RT112 (Figure 3a & 3b), which expands on our previous observations that the p53 gene region is repaired more rapidly than the overall genome following DNA damage [21], [22]. This preferential repair of the p53 gene region compared to both the overall genome and the hTERT gene region was also observed in normal fibroblast cells (Figure 5a and 6a), indicating that repair of strand breaks within, or in the vicinity of, the p53 probe region is carried out quickly (ie within 15 minutes) in a significant number of analysed cells, whereas breaks in or near the hTERT gene region were not. As we have previously explained [22], these changes in hybridisation spot number and location within cells are consistent with our calculations of the likelihood of breaks occurring within (or near) the probed regions during radiation, as well as our understanding of how DNA migrates during the Comet assay. This interpretation is reviewed and supported by Spivak et al. [31] and the findings of Horvathova et al. [32], which suggest that the patterns of migration of domain-specific signals may depend on the localisation of breaks within or around the probed region.

These results mean it is clear that we are able to measure differences in repair of gene regions using the comet-FISH assay, which is not particularly surprising since it is known that DNA repair is not uniform across the genome. For example, it is well established that transcriptionally active regions of the genome are preferentially repaired in contrast to the inactive regions [33]–[35], whilst other studies have used other methods to demonstrate variations in repair rates between different actively transcribed genes [18] and even within the exons of a single active gene [36], [37]. Our study allows us to compare the results from the bladder cancer cell lines (which have active hTERT) with those from normal fibroblasts (which have inactive hTERT). The results show that the preferential repair observed in the cancer cells is not simply a function of transcriptional activity, otherwise we would expect both the p53 and the hTERT gene regions to be preferentially repaired in comparison to overall genome in the cancer cells. Since this is not the case, other contributory factors must be involved in these cells.

Another consideration is that the efficiency of DNA repair may be dependent on the chromatin configuration and nuclear architecture of different regions of the genome [37], [38], a view supported by previous comet-FISH studies from our laboratory [22] and others [39]. Although active genes are normally found in a more open chromatin configuration than inactive regions, thereby enabling easier access for DNA repair proteins, the hTERT gene is known to be embedded in a condensed chromosomal region in various normal, immortal and cancer cell lines regardless of the status of hTERT expression [40]. It has also been suggested that hTERT transcription does not require global chromatin de-condensation but the loss of repressors of hTERT expression [40], [41]. This repressive environment may limit the access to, and the binding of repair proteins to, the sites of damage. Indeed, this would explain why the hTERT gene region exhibits higher levels of damage than the p53 gene region during repair in our experiments and may also go some way to explaining why a number of untreated cells in our experiments showed significant damage in the hTERT gene region. It is possible that even the low amount of background DNA damage in these control cells was sufficient to affect particularly susceptible regions of the genome, such as the hTERT gene region. A final consideration for the high level of damage in the hTERT gene region is its very close location to telomeres at the extreme terminus of chromosome 5p [42]. Using the comet-FISH technique, telomeres were found to be more fragile compared to total DNA to particular cytostatics in both treated and non-treated cells [43]. It has been suggested that the location of most telomeres near the nuclear membrane may offer them a greater chance of migration following DNA damage [44].

Comparison of results from normal fibroblasts with CS fibroblasts presents some intriguing data and invites some speculation on TCR activity in these cells. Normal fibroblasts carry out rapid preferential repair of the active p53 gene region in comparison to the inactive hTERT gene region following γ-radiation exposure (Figure 5a). However, in CS cell lines the active p53 gene region is not preferentially repaired following γ-radiation and repairs at a rate similar to the inactive hTERT gene region and the overall genome (Figures 5b & 5c). Although the comet-FISH assay is not an established method for measuring TCR, it is tempting to propose that these observations reflect the TCR-defective status of CS cells, whereby overall genome repair can be carried out but the rapid preferential repair of actively transcribed genes cannot occur (e.g. in the p53 gene). If this is true, this raises some interesting questions since TCR is not normally associated with the repair of DNA damage induced by ionising radiation. However, it is worth noting that recent evidence has demonstrated that CSA and CSB proteins may have a role to play in DNA repair distinct from NER, since fibroblasts deficient in CSA or CSB have been shown to exhibit sensitivity to ionising radiation [45]–[49]. The results presented here would suggest that fibroblasts defective in CSA or CSB proteins do have reduced ability to repair strand breaks and DNA damage induced by ionising radiation. Whether this is due to defective TCR or not cannot be determined conclusively using the comet-FISH assay, but it would be interesting to see if this method could detect a similar lack of preferential repair in these fibroblasts following UV-radiation, which is more traditionally accepted to be linked to TCR.

It should be noted that, due to the size of the probes and the nature of the comet assay, we are precluded from stating categorically that damage and repair are occurring within a given gene. Rather, the assay enables us to conclude whether or not the damage and repair are occurring in the region including, and surrounding, the gene of interest. However, chromatin structure and rearrangement in the locality of the gene are likely to be important for efficient DNA repair, therefore investigation of DNA regions which include the gene of interest, as well as the individuals genes, is warranted. We also accept that the results probably reflect a number of cells in late S, G2 or mitosis, in which the relevant sequences have been replicated, accounting for those normal cells that show hybridisation spots. However, given the relatively short time-frame employed, this is evidently a small population of cells and would be presumably similar in each sampling. Therefore, we can still make comparison between samples.

In conclusion, we present evidence that the comet-FISH assay can detect differences in DNA repair between two separate gene regions in a variety of cell lines. It may be possible that this approach can be used to evaluate TCR and therefore will merit further investigation. From the results presented in this paper, however, it is clear that this assay is a promising technique that offers great potential for gaining further insight into the process of preferential repair in gene regions within cells. The ability to obtain such information increases the potential value of the Comet-FISH assay as the basis for a predictive test for radiosensitivity in a clinical setting.
